# Tumor-Induced Osteomalacia Mimicking Metastases on Ga-68 DOTATATE Scan: A Rare Pericytic Variant of Phosphaturic Mesenchymal Tumor

**DOI:** 10.1016/j.radcr.2025.11.017

**Published:** 2025-12-04

**Authors:** Laith Abandeh, Troy Hutchens, Manuela Matesan

**Affiliations:** a Department of Radiology, University of Washington, Seattle, WA, USA; b Department of Laboratory Medicine & Pathology, University of Washington, Seattle, WA, USA

**Keywords:** Tumor-induced osteomalacia, Phosphaturic mesenchymal tumor, Fibroblast growth factor 23, Hypophosphatemia, DOTATATE PET/CT, Oncogenic osteomalacia

## Abstract

Oncogenic osteomalacia (OOM), also known as tumor-induced osteomalacia (TIO), is a rare paraneoplastic syndrome caused by excessive production of fibroblast growth factor 23 (FGF23) by mesenchymal tumors. This leads to renal phosphate wasting, hypophosphatemia, decreased 1,25-dihydroxy vitamin D levels, and impaired bone mineralization. Patients often present with progressive muscle weakness, diffuse bone pain, and recurrent fractures, with symptoms frequently preceding diagnosis by several years due to the non-specific nature of hypophosphatemia and its exclusion from routine laboratory panels. We present the case of a 63-year-old man with a seven-year history of progressive musculoskeletal symptoms and multiple fractures. Despite extensive prior evaluations, his diagnosis was delayed until advanced imaging with Ga-68 DOTATATE PET/CT identified a somatostatin receptor-positive lesion in the right superior pubic ramus. Histopathological analysis confirmed a pericytic neoplasm, a rare histologic variant of phosphaturic mesenchymal tumor, consistent with the etiology of OOM. Laboratory evaluation demonstrated profound hypophosphatemia, elevated FGF23 levels, and reduced 1,25-dihydroxy vitamin D. Given the patient’s elevated surgical risk, percutaneous cryoablation of the tumor was performed, resulting in normalization of serum phosphate levels and resolution of clinical symptoms. This case highlights the diagnostic challenges associated with OOM, the utility of somatostatin receptor-based PET/CT in tumor localization, and the importance of considering TIO in patients with unexplained hypophosphatemia and osteomalacia. Prompt recognition and surgical intervention can lead to complete resolution of symptoms and prevent long-term skeletal complications.

## Introduction

Oncogenic osteomalacia (OOM), or tumor-induced osteomalacia (TIO), is a rare paraneoplastic condition that is characterized by bone pain, muscle weakness, and osseous fractures in addition to persistent hypophosphatemia brought on by renal phosphate depletion, normal or low 1,25(OH)2D, and increased or inappropriately normal fibroblast growth factor 23. (FGF23). TIO is caused by tumoral increased production of FGF23, which predominantly operates at the proximal renal tubule to prevent the hydroxylation of 25-hydroxyvitamin D and phosphate reabsorption, which results in hypophosphatemia and, ultimately osteomalacia [1]. Less than 1000 cases of TIO have been documented in the literature, making it a rare condition. True prevalence and incidence statistics, however, are not available. Studies indicate that TIO, which affects both men and women equally and typically manifests between the ages of 40 and 45, is the most prevalent acquired cause of FGF23-mediated hypophosphatemia [1,2].

Given the vague nature of hypophosphatemia's symptoms, it is likely that the condition's actual onset begins several months or even years before overt manifestations like fractures and bone pain. TIO appears to be sporadic and not inherited since no reports have indicated several cases within a single family or a racial or ethnic predilection [3]. Many patients' diagnoses of hypophosphatemia and TIO are delayed because the blood phosphate is excluded from many routine comprehensive chemistry panels [4].

## Case presentation

A 63-year-old male presented to our clinic with complaints of progressive muscle weakness, bone pain, and multiple fractures over the past seven years. The patient had symptoms for two years when he developed stiffness and pain in his left knee. He subsequently developed weakness and pain bilaterally in his thighs, leading him to use a cane and a walker for assistance. The patient also reported chronic fatigue and difficulty with physical activity. His past medical history was significant for hypertension and hyperlipidemia, and he had no family history of bone or metabolic disorders. On examination, the patient was noted to have diffuse muscle weakness and significant pain on palpation of the long bones. He also had multiple fractures, including multiple ribs and long bones. Initial laboratory investigations revealed normal calcium and parathyroid hormone levels. The serum alkaline phosphatase level was elevated (355 U/L), and the serum phosphorus level was low (Table 1).

**Table 1:** Laboratory findings supporting phosphaturic mesenchymal tumor.

Test	Observed value	Reference range	Interpretation
Serum phosphorus	1.1 mg/dL	2.5-4.5 mg/dL	Low—suggestive of phosphate wasting
Serum calcium	Normal	8.5-10.5 mg/dL	Normal
Parathyroid hormone (PTH)	Normal	10-65 pg/mL	Normal
Alkaline phosphatase (ALP)	355 U/L	44-147 U/L	Elevated—marker of increased bone turnover
FGF-23 (C-terminal)	257 RU/mL	<180 RU/mL	Elevated—diagnostic of phosphaturic mesenchymal tumor
1,25-dihydroxy vitamin D	13 pg/mL	20-60 pg/mL	Low—impaired vitamin D metabolism due to FGF-23
24-hour urinary calcium	Low	100-300 mg/24 hr	Low—often seen in OOM
Phosphaturia (fractional excretion of phosphate)	High	<5%	Increased phosphate excretion

He had previously undergone numerous inconclusive diagnostic laboratory tests and imaging studies. His initial workup outside the United States was notable for bilateral femoral neck lesions and asymptomatic rib fractures. An outside Tc-99m MDP bone scan performed there showed multiple areas of increased uptake involving the femoral necks and ribs, findings attributed to fractures and metabolic bone disease. However, the underlying etiology was not established.

**Fig. 1:**
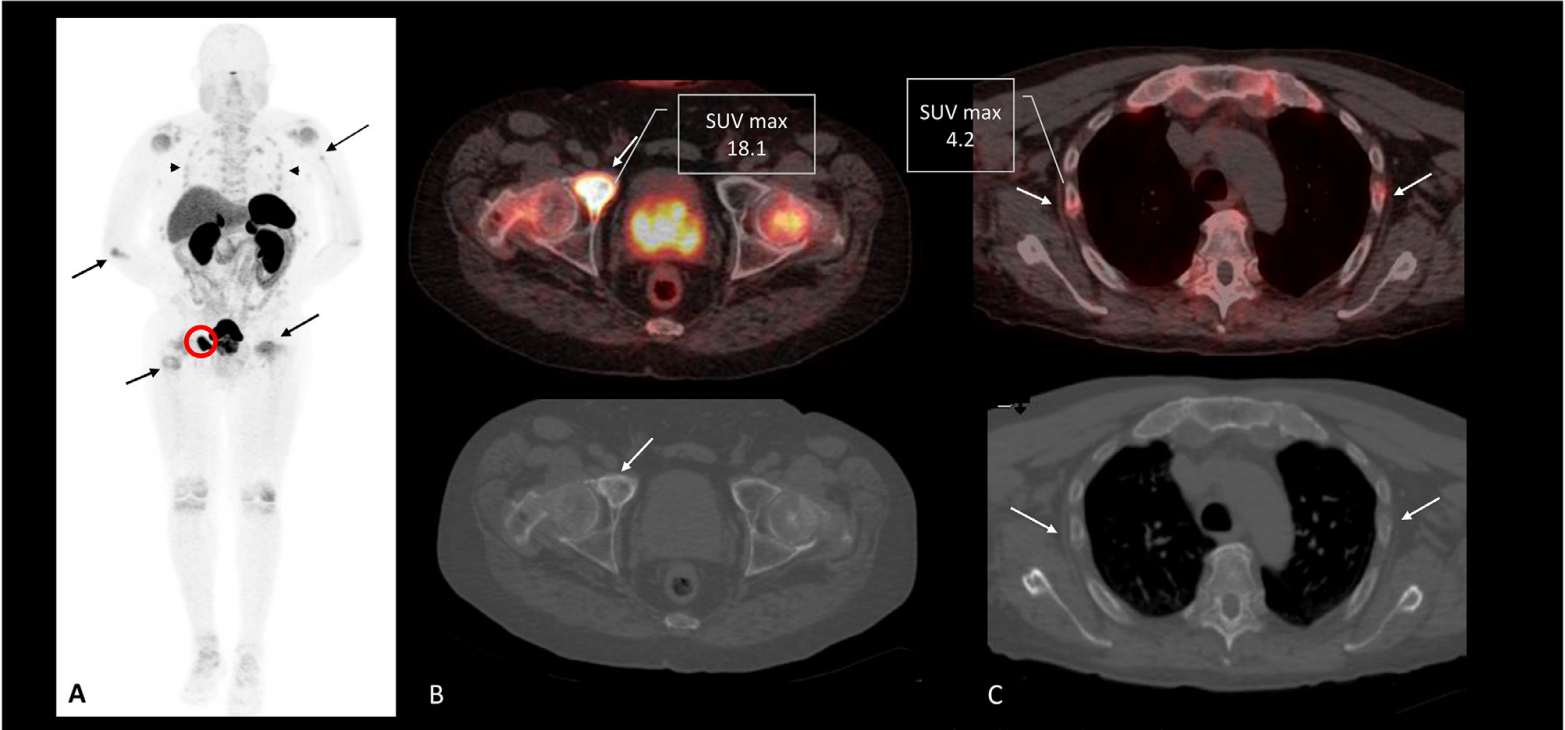
Ga-68 Dotatate PET-CT scan—(A) MIP image demonstrates multiple radiotracer avid looser zones (insufficiency fractures) involving the multiple ribs (arrowhead), left humerus, right ulna, and both proximal femurs (arrows). It also shows a somatostatin-receptor-rich lesion in the right anterior acetabulum (biopsy-proven phosphoturic mesenchymal tumors) with an SUV max of 18.1. (B) PET/CT axial fused and CTAC images show the sclerotic acetabular lesion (PMT) with an SUV max of 18.1. (C) PET/CT axial fused and CTAC images demonstrate the rib fractures (arrows) with SUV max (4.2) much lower than that exhibited by the primary acetabular mass.

After relocation, repeat imaging in the United States again demonstrated multiple fractures. Tc-99m MDP bone scan confirmed bilateral rib and hip fractures, with additional uptake in the knees, ankles, and feet. Subsequent Ga-68 DOTATATE PET/CT localized a somatostatin-receptor–rich lesion in the right superior pubic ramus (Fig. 1).

**Fig. 2:**
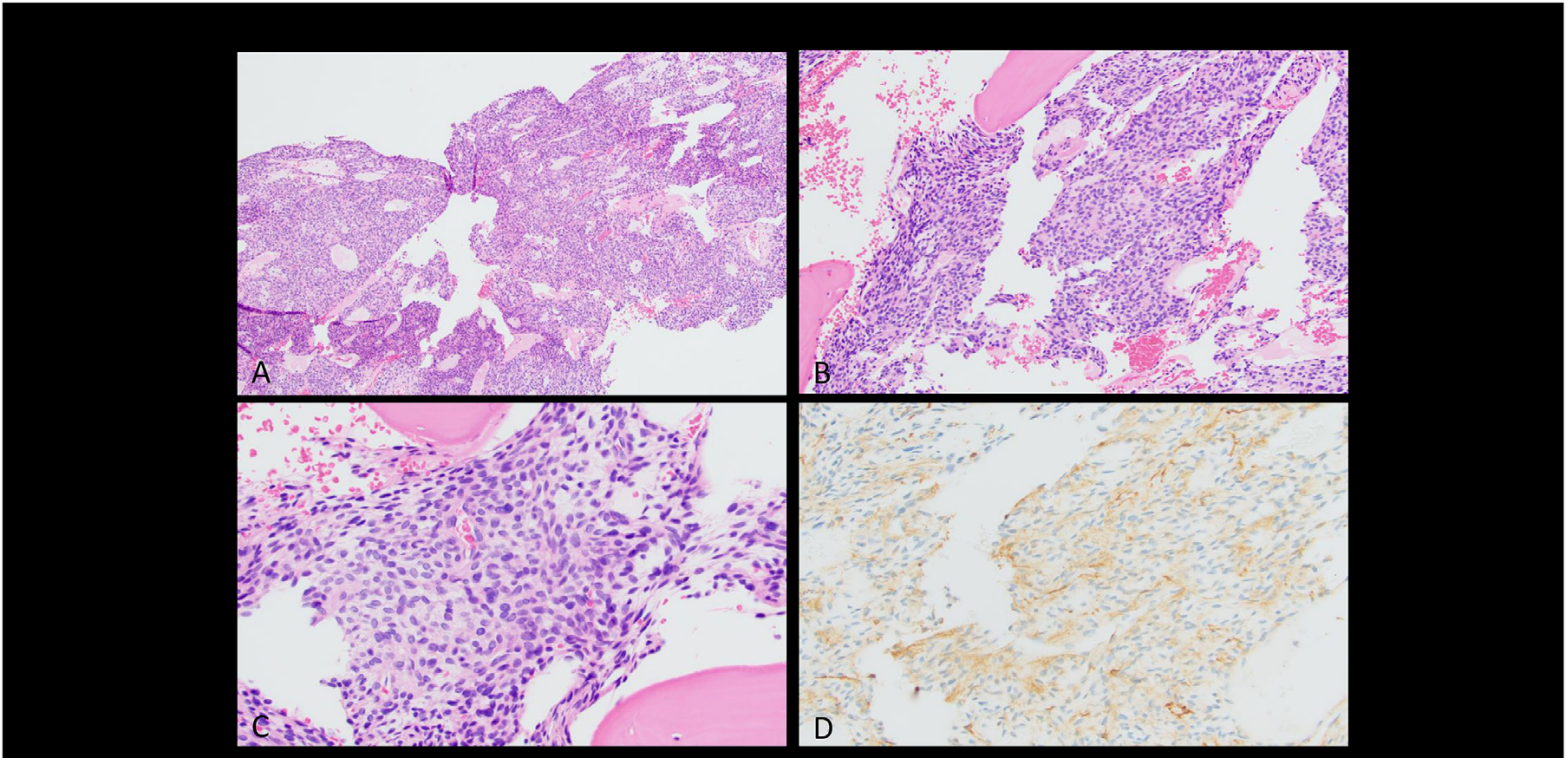
Histopathology and immunohistochemistry of tumor biopsy. *Histopathology and Immunohistochemistry of Tumor Biopsy.* (A-C) Hematoxylin & Eosin (H&E) sections show a bland spindle-cell neoplasm with numerous thin-walled vessels and ovoid nuclei. Cells demonstrate subtle perivascular whirling. (A) Low-power view (original total magnification × 100). (B) Intermediate-power view (original total magnification × 200). (C) High-power view (original total magnification × 400). (D) Immunohistochemistry for SMA shows weak cytoplasmic positivity, supporting pericytic/myoid differentiation (original total magnification × 400).

The patient subsequently had a CT-guided biopsy of the right pelvic bone lesion, and the immunohistochemical stains showed neoplastic cells that were positive for SST2A (diffuse) and weakly positive for SMA. The following stains were negative: PSA, CK7, CK20, PSAP, synaptophysin, CD34, SOX10, ERG, HMB45, OSCAR, desmin, ER and PR. Additional immunohistochemical stains were positive for bcl-2, weakly positive for SMA, and negative for EMA and CD99 (Table 2). The findings were compatible with epithelioid and spindle cell proliferation, consistent with a pericytic neoplasm (Fig. 2).

**Table 2:** Immunohistochemical profile of tumor biopsy.

Marker	Result	Comment
SST2A	Diffusely positive	Supports diagnosis of phosphaturic mesenchymal tumor
SMA	Weakly positive	Pericytic/myoid differentiation
Bcl-2	Positive	Common in mesenchymal tumors
PSA, PSAP	Negative	Rules out prostate origin
CK7, CK20	Negative	Rules out epithelial origin
Synaptophysin	Negative	Neuroendocrine tumor less likely
CD34, ERG	Negative	Endothelial markers-Fnot vascular tumor
Desmin, HMB45, SOX10	Negative	Rules out myogenic, melanocytic tumors
EMA, CD99	Negative	Excludes Ewing’s sarcoma, epithelial tum

**Fig. 3:**
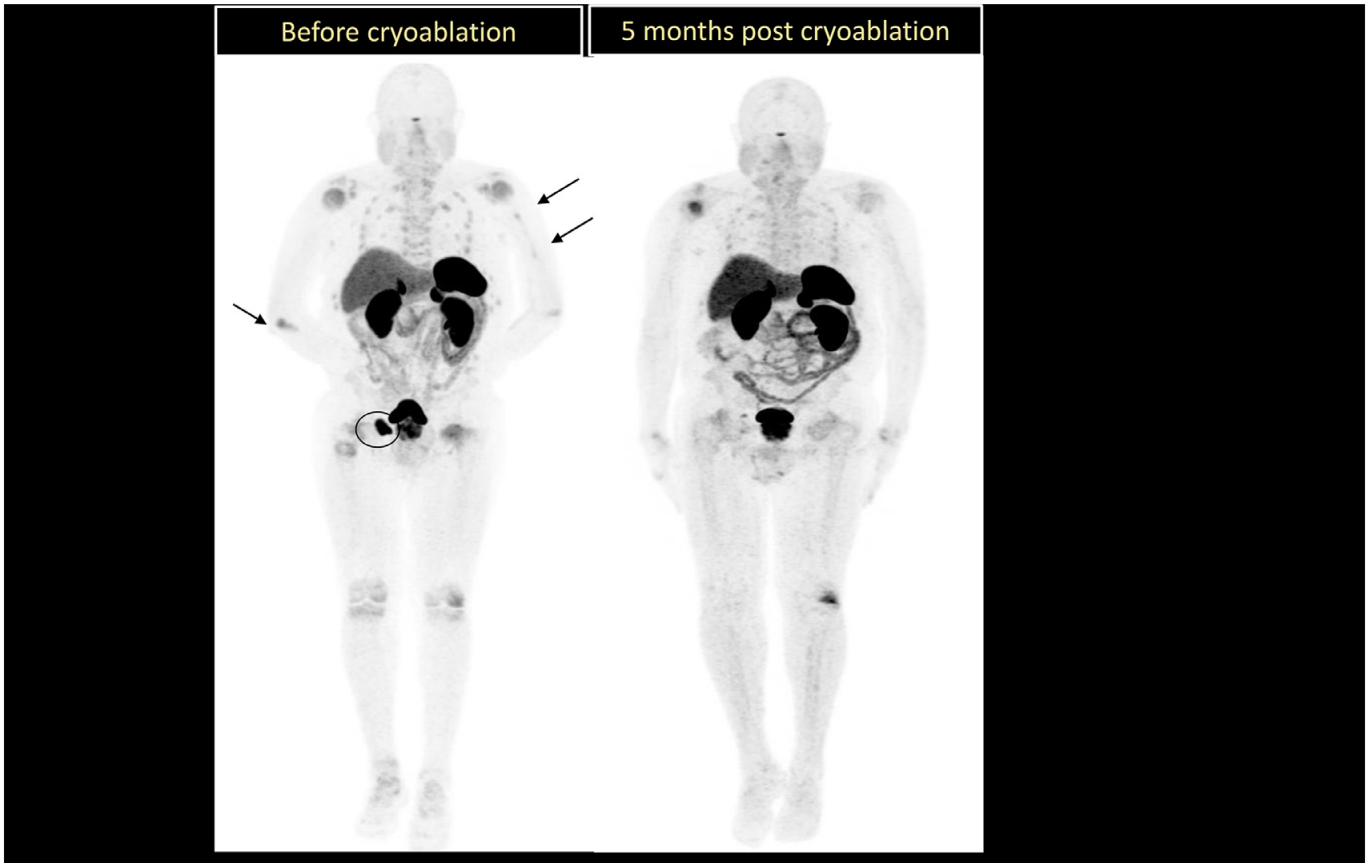
Ga-68 Dotatate MIP images before and after cryoablation of the culprit lesion in the right acetabulum, showing complete resolution of the radiotracer avid fractures/Looser’s zones in the ribs and extremities. This was also associated with clinical and biochemical remission (normalization PTH and phosphorus levels).

Subsequent workup by the endocrinologist was notable for elevated FGF-23 activity (257 RU/mL), hypophosphatemia (1.1 mg/Dl), hypophosphaturia, low 1,25-dihydroxy vitamin D (13 pg/mL), and low 24-hour urinary calcium levels (Table 1).

Based on the clinical presentation, laboratory investigations, and histopathology, an oncogenic osteomalacia (OOM) diagnosis was made. Given the patient’s elevated surgical risk, percutaneous cryoablation of the right acetabular tumor was performed. His symptoms improved significantly following the procedure, with normalization of serum phosphate levels and resolution of bone pain and muscle weakness. A follow-up Ga-68 DOTATATE PET scan was subsequently performed (Fig. 3).

## Discussion

Due to its rarity and vague clinical presentation, OOM is frequently challenging to diagnose. The pathophysiology of OOM involves the production of FGF23 by mesenchymal tumors. Excessive FGF23 leads to increased urinary phosphate excretion and decreased serum phosphate levels, resulting in hypophosphatemia and osteomalacia. Phosphaturic mesenchymal tumors (PMTs) are typically small and difficult to localize, but they commonly express somatostatin receptors [5]. They can arise in virtually any bone or soft tissue and are most common in middle-aged individuals. Histologically, PMTs show spindle-cell proliferation and variable “smudgy” calcified matrix. While a minority may demonstrate malignant features, most are benign and rarely metastasize [6].

Somatostatin receptor (SSTR) PET/CT imaging, such as Ga-68 DOTANOC and DOTATATE PET/CT, is considered the first-line modality for localizing PMTs in suspected TIO [7]. Typically, culprit lesions demonstrate uptake much higher than insufficiency fractures or Looser’s zones. In this case, the SUVmax of the tumor (18.1) was markedly higher than that of the fractures (4.2), providing a clear distinction between primary pathology and skeletal complications. Such a strong imaging contrast has been only rarely described in the literature and underscores the utility of DOTATATE PET/CT in differentiating culprit tumors from secondary skeletal changes.

Another unique feature of this case is the prolonged diagnostic delay of seven years, significantly longer than the average 2-5 years commonly reported. This delay contributed to extensive skeletal involvement, including multiple rib and long bone fractures, highlighting the morbidity associated with late recognition of hypophosphatemia. Additionally, histopathological analysis revealed a pericytic variant of PMT, a rare histological subtype that broadens the known morphological spectrum of these tumors.

The mainstay of treatment for OOM is surgical resection of the causative tumor, which is usually curative. In cases where surgery poses heightened risk, minimally invasive alternatives such as percutaneous cryoablation may be effective. In this case, cryoablation led to rapid biochemical normalization and resolution of symptoms. Hypophosphatemia and osteomalacia may occasionally be managed temporarily with phosphate supplementation and calcitriol in unresectable cases or while awaiting surgery [8]. Long-term follow-up is required to monitor for recurrence [8]. Importantly, this case demonstrates that even after years of morbidity, timely localization with advanced imaging and definitive surgical resection can result in complete clinical, biochemical, and radiographic remission.

## Patient consent

A consent from the patient has been obtained for publication. The authors affirm that that all images and clinical details have been fully anonymized to prevent patient identification.
